# Remimazolam Attenuates LPS-Derived Cognitive Dysfunction via Subdiaphragmatic Vagus Nerve Target α7nAChR-Mediated Nrf2/HO-1 Signal Pathway

**DOI:** 10.1007/s11064-024-04115-x

**Published:** 2024-03-12

**Authors:** Zhan Zhou, Ying Yang, Yi Wei, Yubo Xie

**Affiliations:** 1 Department of Anesthesiology, The First Affiliated Hospital of Guangxi Medical University, Nanning, 530021 China; 2 Guangxi Key Laboratory of Enhanced Recovery after Surgery for Gastrointestinal Cancer, The First Affiliated Hospital of Guangxi Medical University, Nanning, 530021 China

**Keywords:** Remimazolam, Lipopolysaccharide, Microglia, Neuroinflammation, Cognitive dysfunction

## Abstract

Sepsis-induced neuroinflammation is significantly associated with sepsis-related brain dysfunction. Remimazolam is a novel ultra-short-acting benzodiazepine anesthetic with multiple organ protective effects. However, it is unknown whether remimazolam can ameliorate LPS-induced brain impairment. In this study, Lipopolysaccharide (5 mg/kg, LPS) severely impaired Sprague-Dawley rats spatial learning ability, memory, and cognitive function. However, remimazolam treatment showed a protective effect on LPS-induced cognitive dysfunction. Remimazolam partly reversed LPS-induced splenomegaly, decreased serum cytokine expression, suppressed hippocampal M1 microglial activation, and mitigated oxidative stress injury and neuroinflammation. Electroacupuncture (EA) or PNU282987 treatment improved LPS-induced cognitive dysfunction and also significantly inhibited neuroinflammation and systemic inflammation. However, MLA, ML385, or subdiaphragmatic vagus nerve (SDV) treatment abolished the protective effects of remimazolam. Further mechanistic studies showed that remimazolam induces protective effects by activating subdiaphragmatic vagus nerve target α7nAChR-mediated Nrf2/HO-1 signaling pathway. These results demonstrate that remimazolam can up-regulate α7nAChR, Cyto-Nrf2, HO-1, and cognitive-related (CREB, BDNF, PSD95) protein expressions, suppress M1 microglia, ameliorate neuroinflammation or systemic inflammation, and reverse cognitive dysfunction. Therefore, this study provides insight into a new therapeutic target for the treatment of sepsis-induced cerebral dysfunction.

**Graphical Abstract:**

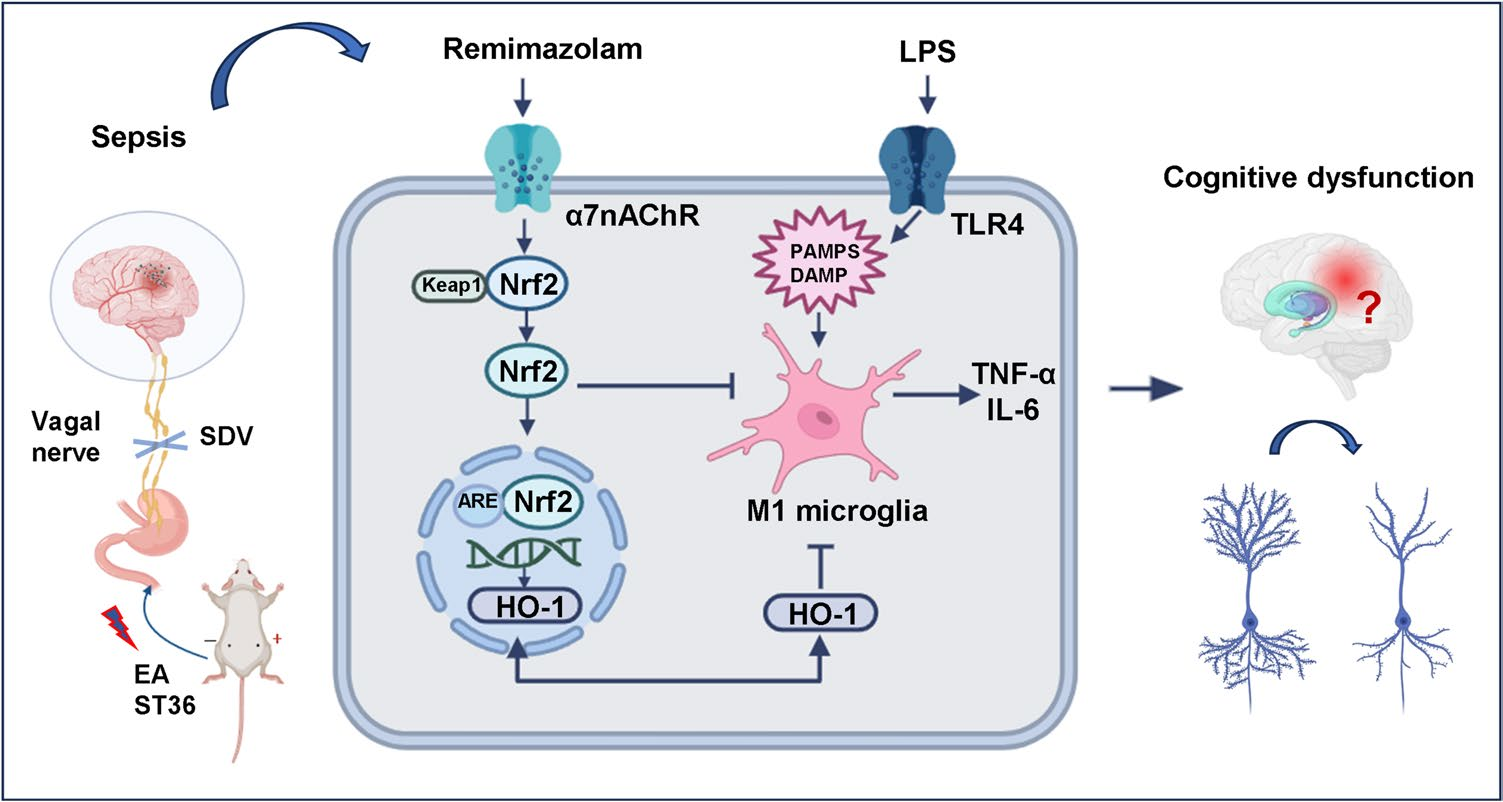

## Introduction

Sepsis is a dysregulated host response to infection that results in life-threatening organ dysfunction. Sepsis affects more than 30 million people worldwide [1]. Inflammation is strongly associated with sepsis. Besides, sepsis patients can experience impairments in long-term cognitive after being discharged from the hospital [2], thus severely reducing their quality of life, increasing mortality rates, and increasing economic pressure on society, families, and individuals [3]. The current management of acute sepsis-associated brain injury mainly focuses on treating sepsis as the underlying disease, with no specific treatments to address brain dysfunction.

Central nervous system (CNS) is usually impacted at the early stage of sepsis. Sepsis triggers the host immune response causing vascular endothelial damage, thus breaking the blood-brain barrier (BBB), allowing the entry of peripheral immune cells into the brain, which triggers or exacerbates glial cell activation and neuroinflammation [4]. Microglial activation plays essential roles in immune surveillance, synaptic plasticity regulation, and maintaining dynamic homeostasis within the CNS. Microglia activation under pathological conditions induces synaptic loss, neuron dysfunction, and neural circuit disruption [5]. Therefore, microglia, as the first line of defense against pathogens, are critical for maintaining CNS homeostasis [6].

Remimazolam is a novel benzodiazepine anesthetic agent that binds to the benzodiazepine on the GABA receptors. Besides, remimazolam has been widely used for general anesthesia and ICU sedation [7]. Furthermore, remimazolam has protective effects on multiple organs, such as the brain, liver, and lung. Remimazolam can effectively improve neurological dysfunction after cerebral ischemia-reperfusion injury in rats, mitigate sepsis-associated acute liver injury, and increase the survival rate [8–11]. However, it is unknown whether remimazolam has a protective effect on sepsis-associated neurological dysfunction.

In this study, remimazolam suppressed LPS-induced inflammatory cascade generated by microglia activation, thus attenuating neurological damage and cognitive dysfunction. Further results suggested that remimazolam can induce its protective effects through the activation of subdiaphragmatic vagus nerve target α7nAChR-mediated Nrf2/HO-1 signaling pathway. Therefore, in vivo models treatment with or without MLA, PNU282987 or ML385 were used to test α7nAChR-mediated Nrf2/HO-1 signalling pathway in the protective effects of remimazolam via Golgi staining, Morris water maze, Hematoxylin-eosin (HE), and Nissl staining, immunofluorescence, and western blot. The role of the subdiaphragmatic vagus nerve integrity in remimazolam reducing brain dysfunction in sepsis was also evaluated by EA or SDV operation.

## Materials and Methods

### Animals

A total of 150 male Sprague-Dawley (SD) rats were sourced from the Laboratory Animal Center of Guangxi Medical University (Nanning, China). The rats were acclimated in a controlled temperature (25 ± 2 °C) with relative humidity 60 ± 5% and 12:12-h light/dark cycle for a week before the experiments. Also, the rats had free access to food pellets and water. The animal experiments followed the guidelines for the Care and Use of Laboratory Animals and were approved by the Experimental Animal Committee of Guangxi Medical College (Permission number: 202109015).

### Model Establishment and Grouping

**Fig. 1:**
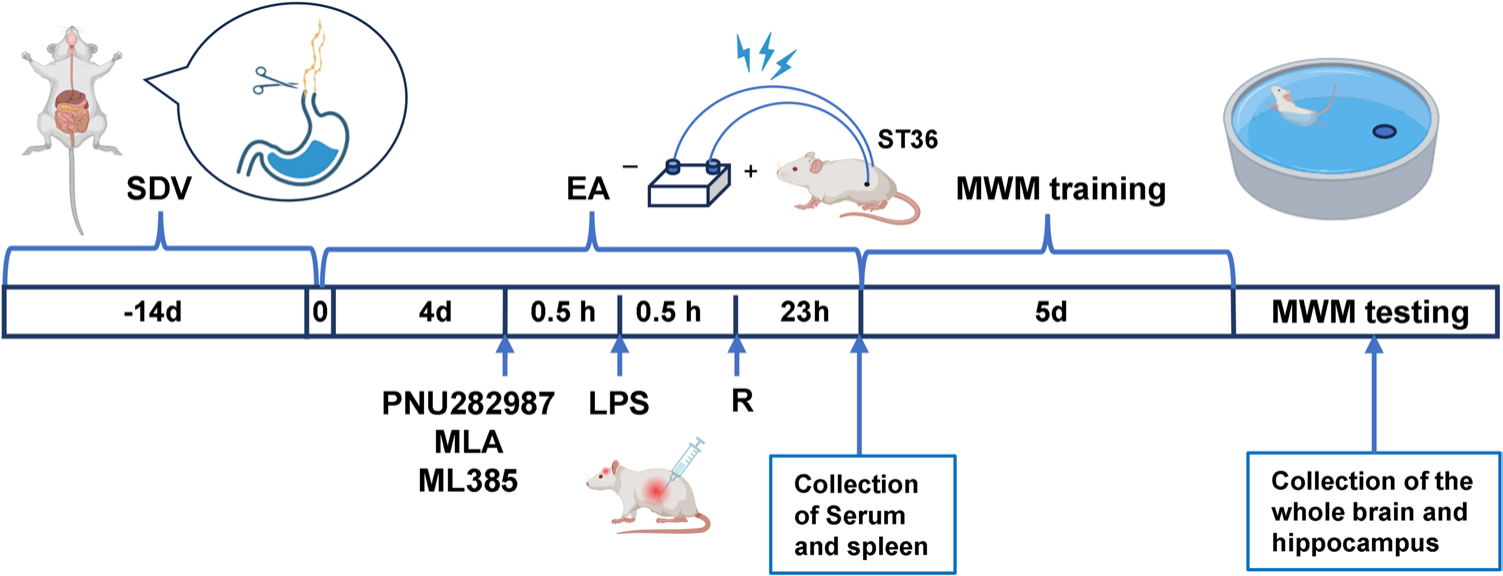
Schematic flowchart of the experiment. The experiment was divided into three parts according to the experimental design. The first phase describes the brain-protective effect of remimazolam. Briefly, control and LPS groups were intraperitoneally (i.p) treated with saline or LPS (5 mg/kg). Remimazolam (10 mg/kg) or saline was intravenously injected 30 min after LPS injection. The second phase involved the investigation of the role of α7nAChR target and Nrf2/HO-1 signaling pathway in remimazolam treatment. Briefly, MLA (3 mg/kg), ML385 (30 mg/kg), or PNU282987 (10 mg/kg) were administered intraperitoneally 30 min before LPS infection. The third phase involved activation of the cholinergic pathway through surgery of SDV or sham rats two weeks before modeling. EA was also performed for five consecutive days. Created with BioRender.com

The animals were randomly assigned to 11 groups (*n* = 12/group, *n* = 6 per cage). The sepsis rat model was established via intraperitoneal injection of 5 mg/kg LPS [12], followed by intravenous injection of remimazolam (10 mg/kg; Jiangsu; China) [8] or 0.9% saline. Notably, α7nAChR agonist (PNU282987, 10 mg/kg, Sigma, USA) [13], Nrf2/HO-1 inhibitor (ML385, 30 mg/kg, Selleck, USA) [14], and α7nAChR antagonist (MLA, 3 mg/kg, abcam) [15] were dissolved in normal saline with 2% DMSO and administered intraperitoneally 30 min before LPS treatment. The SDV or Sham rats underwent surgery two weeks before modeling. EA at ST36 was performed for five consecutive days to activate the cholinergic pathway. The treatment in each group was terminated at the same time, and serum was collected from the tail vein 24 h after LPS injection. The rats underwent behavioral tests, then anesthetized for rapid removal of the whole brain and spleen. The samples were stored at − 80 °C or fixed with 4% paraformaldehyde. The experimental unit was described by one single animal. Moreover, there were no inclusion and exclusion criteria. The groupings were only known to the principal investigator. The order of treatments, measurements, and caging of the animal were randomized to minimize confounding factors (Fig. 1).

### Electroacupuncture (EA)

Zusanli (ST36) acupoints were located 5 mm below the knee joint, 1–2 mm lateral to the anterior tibial tuberosity. Briefly, acupuncture needles (0.16 × 7 mm) were inserted 3 mm into the bilateral ST36 acupoint. EA apparatus (Hwato, Suzhou, China) was connected (0.5 mA and 15 Hz) for 30 min once daily for five consecutive days to stimulate cholinergic anti-inflammatory pathways [16].

### Subdiaphragmatic Vagotomy (SDV)

SDV was induced as previously described [17]. Briefly, gastroesophageal junction was isolated from the anterior and posterior vagus nerves, then ligated with a surgical suture and cut between the ligations. The peritoneal cavity was irrigated with saline, the abdomen was sutured. The sham operation did not transect the vagus nerves. The animals were kept in a single cage after the operation and recovered for two weeks.

### Golgi Staining

Golgi staining was performed as previously described [18]. The samples were observed with Nikon microscope (Nikon, Eclipse Ci-L, Japan). The density of dendritic spines was quantified by counting spines in 30–90 μm length of secondary or tertiary branches of dendrites. Dendritic spine density was determined using Image-Pro Plus 6.0 and ImageJ 1.5.1 software.

### Hematoxylin-Eosin (HE) and Nissl Staining

Hematoxylin and eosin staining (Solarbio, China) and Nissl staining (Beyotime, China) were performed separately following the kit instructions. The morphology of hippocampal was observed under a positive light microscope (Olympus, Japan).

### Immunofluorescence

Sections were routinely dewaxed and dehydrated. The sections were permeated with 0.5% Triton X-100 after repair of antigen, then incubated with 10% goat serum for 1 h. The samples were incubated with primary antibodies, rat anti-iNOS (1:200, Servicebio, China), and mice anti-Iba-1 (1:200, Servicebio, China) at 4 °C overnight. The samples were also incubated with Alexa Fluor 488 conjugated Goat Anti-rabbit IgG (H^+^L) (1:500, Servicebio, China) and Cy3 conjugated Goat Anti-mouse IgG (H^+^L) (1:300, Servicebio, China) secondary antibodies for 1 h. The sections were also incubated with DAPI for 5 min and visualized via fluorescence microscopy (Leica DM IL LED microscope, Wetzlar, Germany). Eight randomly selected fields were used to calculate the average fluorescence intensity of iNOS and Iba-1 via ImageJ 1.5.1 software.

### Enzyme-Linked Immunosorbent Assay (ELISA)

First, hippocampus tissues were homogenized in 0.9% saline, then centrifuged at 12,000 rpm and 4 °C for 15 min. Blood was centrifuged at 3000 g and 4 °C for 5 min to isolate serum and stored at − 80 °C. The concentrations of TNF-α (FineTest, China) and IL-6 (FineTest, China) were measured at 560 nm using microplate reader (ELX450 Bio-Tek), following the manufacturer’s protocol.

### SOD and MDA Measurement

The hippocampus supernatant SOD and MDA contents were assessed via SOD (Jiancheng, Nanjing, China) and MDA kits (Jiancheng, Nanjing, China), following the manufacturers’ instructions. A microplate reader (Bio-Tek, Winooski, VT, USA) was used to measure absorbance at 532 nm for MDA and 450 nm for SOD.

### Morris Water Maze (MWM)

The learning and memory abilities of rats were assessed with Morris water maze (Viewer, German, Biobserve GmbH). The first day of training was dedicated to adapting the animal to the aquatic labyrinth, except for rats with visual deficits. The positional navigation training was performed once in each quadrant for five consecutive days after 24 h of LPS injection. The time taken by the rat to find the platform as escape latency was then recorded. The rats were guided to the platform for 20 s if they could not find the platform, otherwise the escape latency of the rat was 90 s. The platform was removed on the 6th day to record the (90 s) rats in the target quadrant and the frequency of rats crossing the target quadrant [19].

### Molecular Docking

Docking studies were performed to calculate the binding affinities of remimazolam at the α7nAChR binding site using AutoDock Vina (AutoDockTools-1.5.6). The 2D/3D structures of drugs were obtained from PubChem (SDF format), then structured and energy minimized by ChemBioDraw 3D software. Crystal structures were obtained from Protein Data Bank (PDB, https://www.rcsb.org/). The crystal structure was developed using AutoDock Vina, including removing water molecules and adding hydrogens, then saved this structure in pdbqt format. Molecular docking was performed using the AutoDock-Vina program after a grid was positioned on the active site region of the protein. The binding affinity was calculated as Kcal/mol. The resulting models were visualized using PyMOL 2.3.0 and BIOVIA Discovery Studio 2016 [20].

### Western Blot

Hippocampus tissues were lysed using tissue lysis buffer for protein extraction. Protein concentration was quantified via the BCA method (Beyotime, China), then separated by 12% SDS-PAGE gels (Meilunbio, China) and transferred to PVDF membranes (0.22 μm, Millipore, America). The membranes were blocked with 5% non-fat milk powder for 1 h, then incubated with primary antibodies α7nAChR (1:1000, Affinity Biosciences, China), Nrf2 (1:1000, CST, China), HO-1 (1:1000, CST, China), CREB (1:1000, Affinity Biosciences, China), BDNF (1:1000, abcam, China), PSD95 (1:1000, CST, China) at 4 °C overnight. The membranes were washed thrice with TBST for 5 min, then incubated with goat anti-rabbit IgG (H^+^L) secondary antibody (1:10000, Thermo Fisher, America) at 4 °C for 1 h. Finally, the membranes were scanned using Odyssey system (LI-COR, America).

### Statistical Analysis

GraphPad Prime 9.5 software was used for all statistical analyses. Data with normal distribution were expressed as the *mean ± SME*. One-way analysis of variance (ANOVA) test and Tukey test were used to compare groups. *P* < 0.05 was considered a significant level.

## Results

### Remimazolam Suppresses LPS-Induced Inflammation and M1 Microglial Activation

**Fig. 2:**
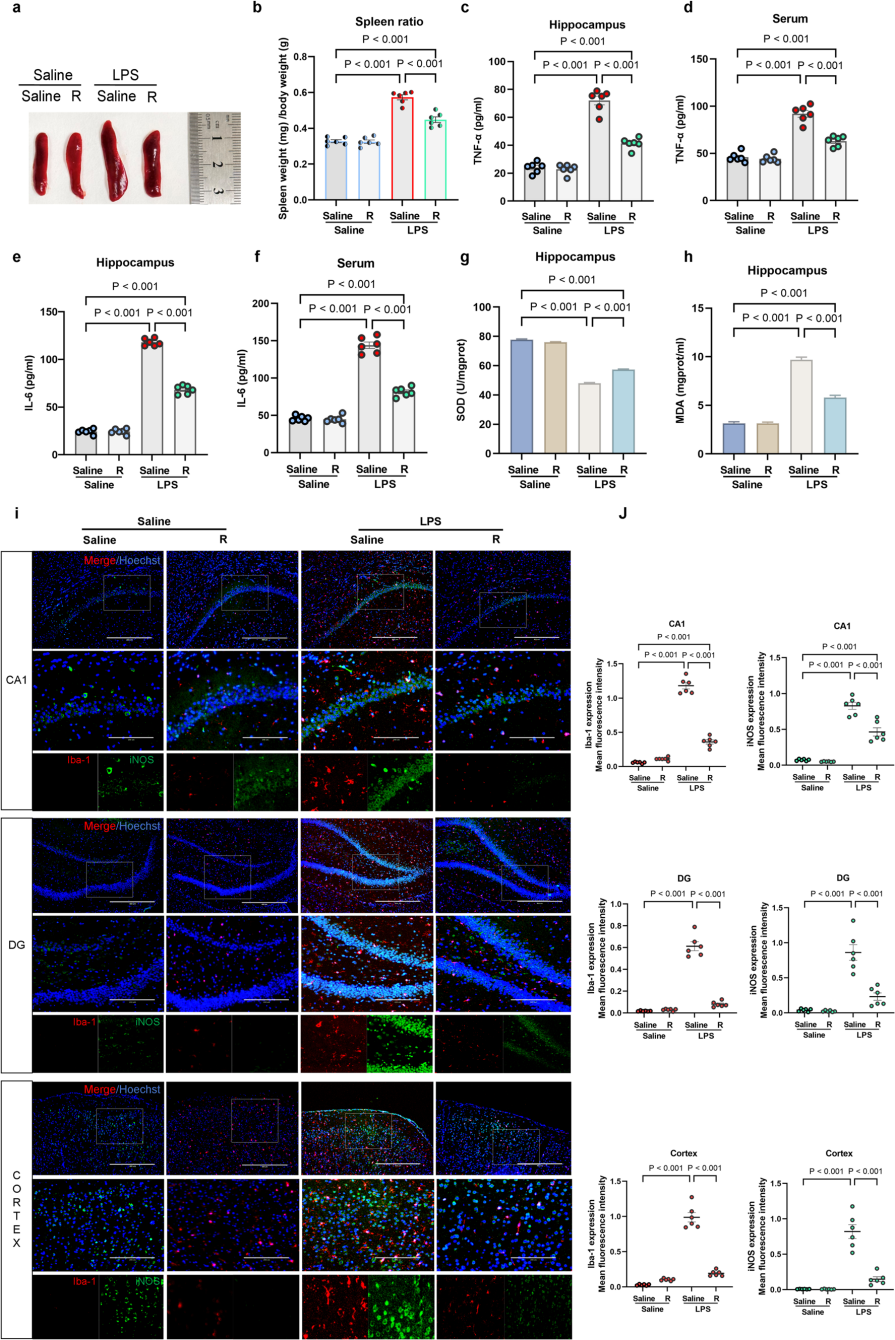
Remimazolam suppresses LPS-induced inflammation and M1 microglial activation **a** Representative images of the spleen. **b** Ratio of spleen weight/body weight. **c** TNF-α expression in hippocampus. **d** Serum TNF-α levels. **e** IL-6 expression in hippocampus. **f** Serum IL-6 levels. **g** SOD activity in hippocampus. **h** MDA content in hippocampus. **i** Immunofluorescence showing the activation of microglia in hippocampus (Red: Iba-1; Green: iNOS; Blue: DIPA; bar: 100 μm). **j** Iba-1 and iNOS expression levels. Data are expressed as *mean ± SEM*, *n* = 6/group

The spleen size, inflammatory factors, and microglial activation were assessed to investigate the anti-inflammatory effects of remimazolam on LPS. Compared with the saline group, LPS significantly increased spleen size, as reflected by the spleen weight/body weight ratio (Fig. 2a, b). Furthermore, LPS increased the release of pro-inflammatory factors TNF-α and IL-6 in serum and hippocampus (Fig. 2c-f). However, remimazolam treatment significantly attenuated the above effects. In addition, remimazolam reversed LPS-induced downregulation of SOD and reduced MDA generation (Fig. 2g, h). Meanwhile, remimazolam suppressed LPS-induced M1 microglial activation in CA1, CA3, and DG regions of the hippocampus and decreased Iba-1 and iNOS expression levels (Fig. 2i, j). These results show that remimazolam can significantly suppress M1 microglial activation and reduce systemic inflammation and neuroinflammation in sepsis rats.

### Remimazolam Attenuates LPS-Induced Cognitive Impairment

**Fig. 3:**
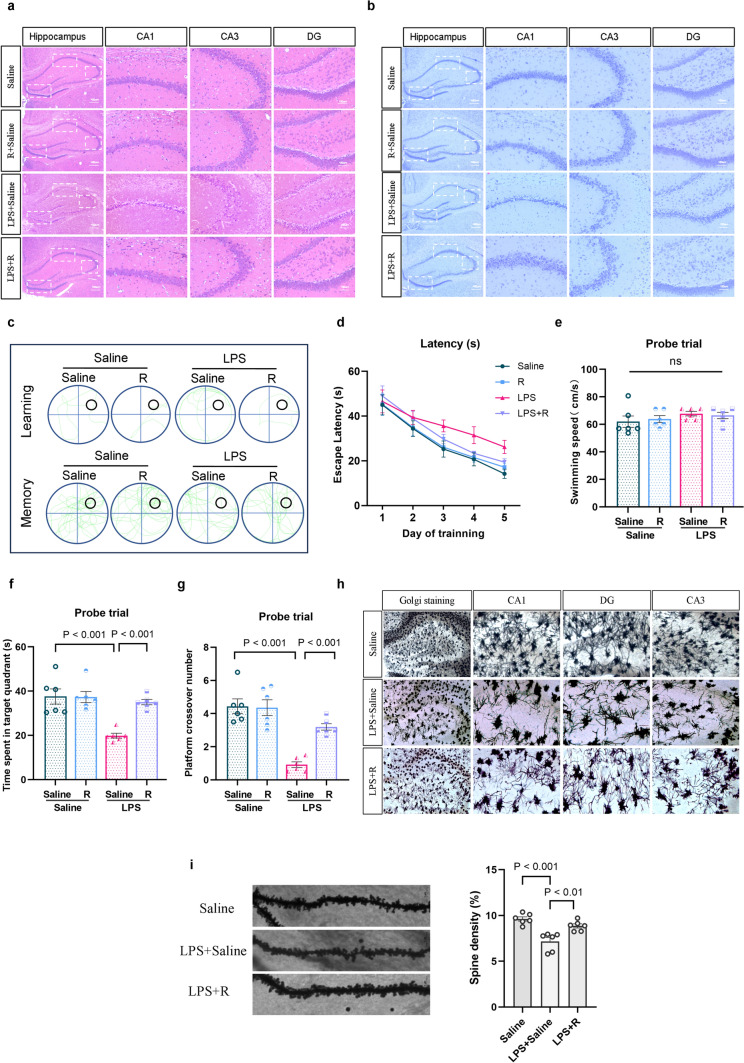
Remimazolam attenuates LPS-induced cognitive impairment **a** HE staining of hippocampus ($\times$200). **b** Nissl-stained hippocampus. **c** The swim tracks of the rat during Morris water maze. **d** Escape latency to the platform during spatial working memory testing on days 1, 2, 3, 4 and 5. **e** Swimming speed during probe testing. **f** Platform-site crossings during probe testing. **g** The time traveled in the target quadrant during probe testing. **h** Representative images of hippocampus via Golgi staining (×200). **i** Representative images of dendrites in neurons of hippocampus (×1000) (The dendritic spine density = the number of spines /the length of dendrites* 10). Data are expressed as *mean ± SEM*, *n* = 6/group

The pathological changes of rat brain tissue and neuronal damage were assessed using HE staining and Nissl staining. LPS induced neuronal nuclear pyknosis, cellular vacuolization, and disordered arrangement. However, remimazolam treatment exhibited a restorative effect on neuronal damage (Fig. 3a, b). Furthermore, Morris water maze test was performed to determine whether remimazolam treatment can attenuate LPS-induced cognitive impairment. The rats were trained to learn a fixed spatial target location in the maze during the positioning navigation trial for five days (Fig. 3c). LPS treatment significantly increased the escape latency (Fig. 3d). Although the swimming speed was not significantly different among the groups (Fig. 3e), LPS decreased the number of platform crossings and the time spent in the target quadrant during the final (probe) trial (Fig. 3f, g). However, remimazolam attenuated the LPS-induced effects. In addition, remimazolam significantly attenuated LPS-induced decrease in dendritic branch points and dendritic spine density (Fig. 3h, i). These results indicate that remimazolam can ameliorate LPS-induced learning and memory deficits by alleviating the changes in synapse and dendritic spine density and neuronal damage.

### The Roles of α7nAChR in Remimazolam Amelioration LPS-Induced Brain Injury in Rats

**Fig. 4:**
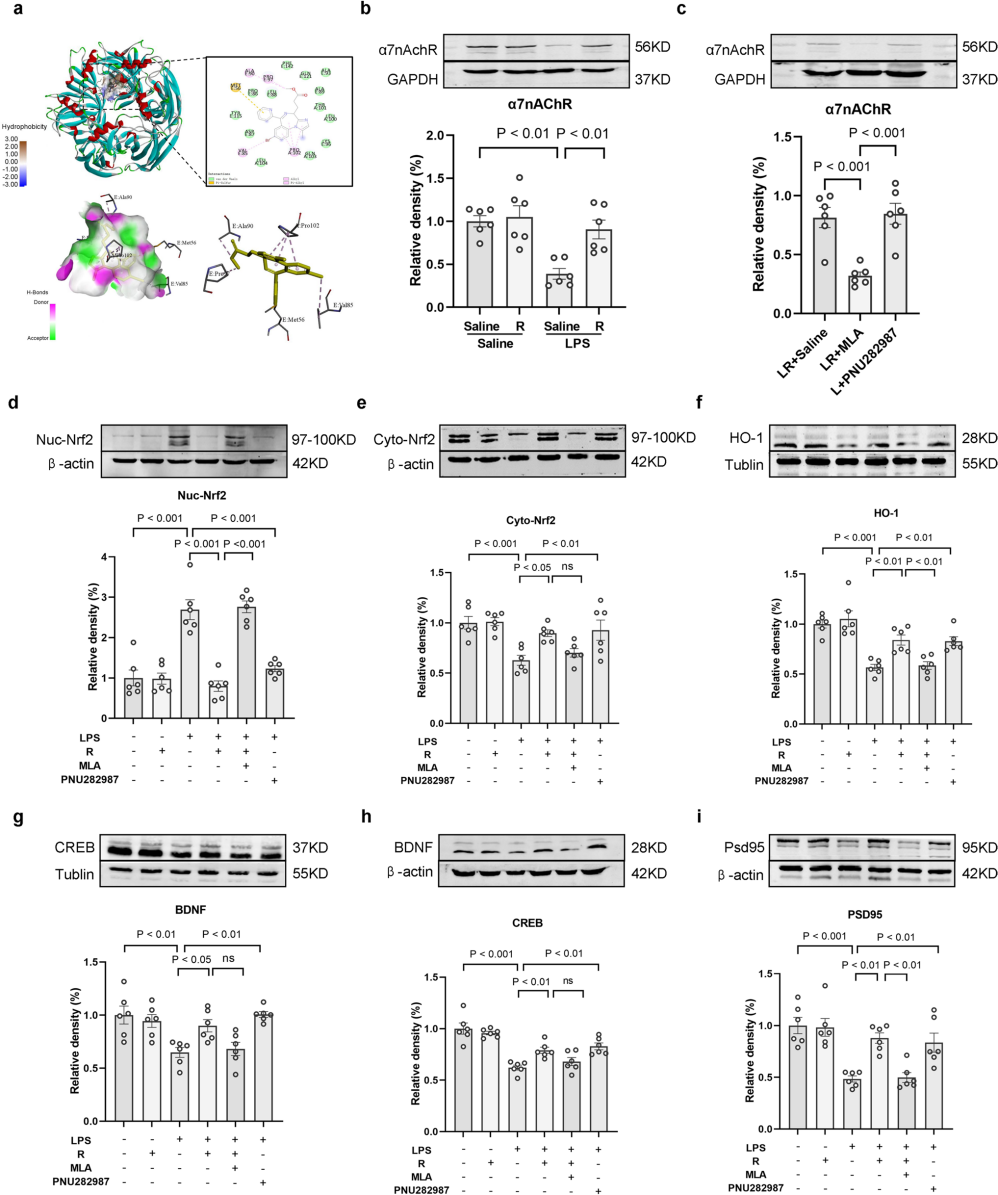
The roles of α7nAChR in remimazolam amelioration LPS-induced brain injury in rat **a** The predicted binding modes of remimazolam with α7nAChR. **b**, **c** Western blot analysis of α7nAChR in hippocampus. **d** Western blot analysis of Nuc-Nrf2 in hippocampus. **e** Western blot analysis of Cyto-Nrf2 in hippocampus. **f** Western blot analysis of HO-1 in hippocampus. **g** Western blot analysis of CREB in hippocampus. **h** Western blot analysis of BDNF in hippocampus. **i** Western blot analysis of PSD95 in hippocampus. Data are expressed as *mean ± SEM*, *n* = 6/group

α7nAChR plays an important role in brain development and synaptic plasticity, as well as in learning and memory [21]. Herein, the binding ability of remimazolam with α7nAChR was evaluated using molecular docking studies to confirm if α7nAChR is a potential target of remimazolam. The best binding energy (kcal/mol) of the compound and target was − 8.2 kcal/mol, indicating a stronger binding in the ligand-receptor complex (Fig. 4a). Western blot analysis showed that LPS significantly decreased the expression level of α7nAChR in the hippocampus. However, remimazolam reversed the above effect (Fig. 4b). The effect of α7nAChR inhibitor MLA and α7nAChR agonist PNU282987 was examined to further assess whether α7nAChR is a target for remimazolam during mitigation of LPS-induced cognitive dysfunction. Compared with LPS injection alone, PNU282987 or remimazolam treatment significantly up-regulated α7nAChR, Cyto-Nrf2, and HO-1 expression and cognitive-related proteins (CREB, BDNF, and PSD95) but downregulated Nuc-Nrf2. Compared with LR group, MLA treatment significantly attenuated the protective effect of remimazolam (Fig. 4c–i).

### α7nAChR-mediated Nrf2/HO-1 Signal Activation Exerts Anti-Inflammatory Effects

**Fig. 5:**
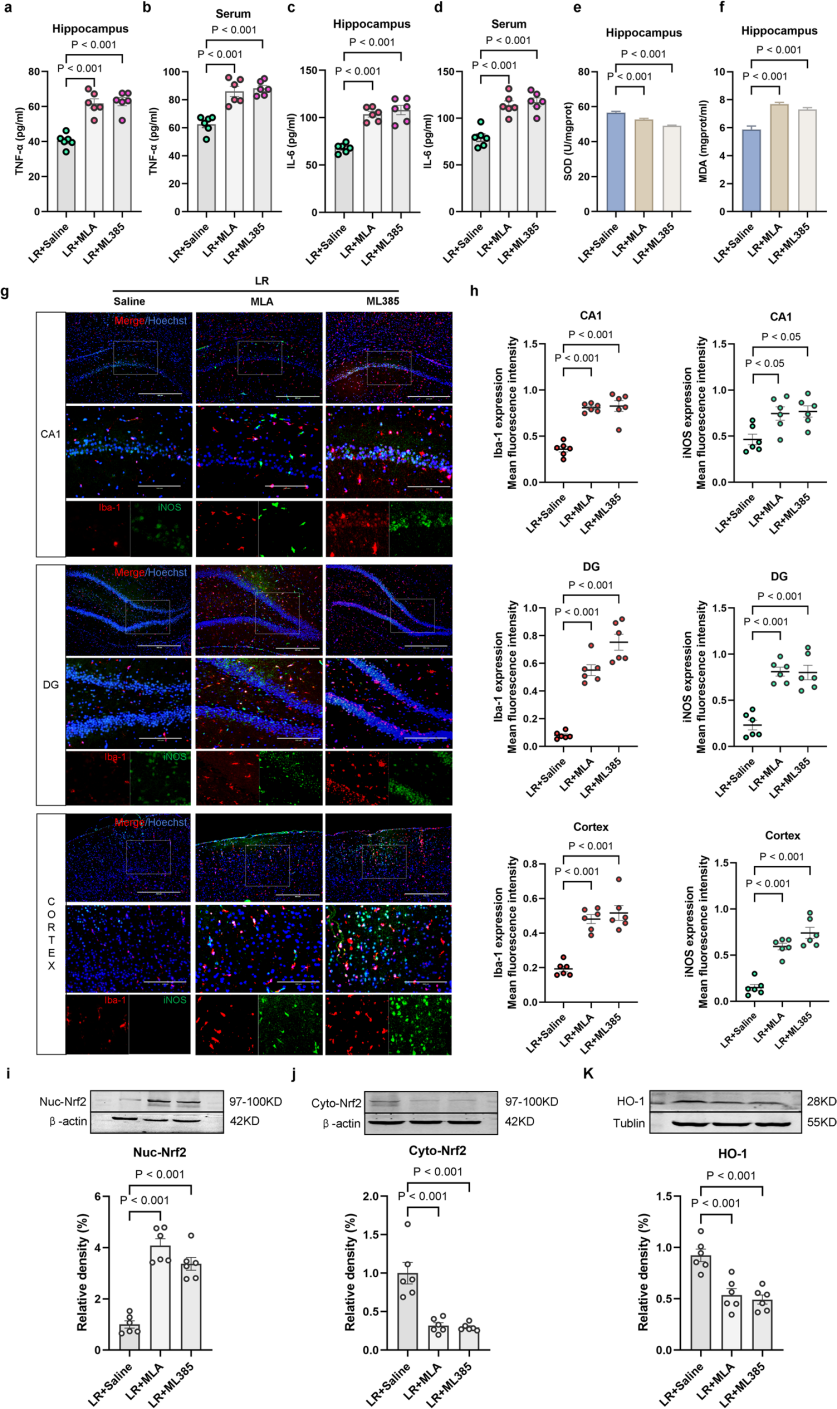
α7nAChR-mediated Nrf2/HO-1 signal activation exerts anti-inflammatory effects **a** TNF-α expression in hippocampus. **b** Serum TNF-α levels. **c** IL-6 expression in hippocampus. **d** Serum IL-6 levels. **e** SOD activity in hippocampus. **f** MDA content in hippocampus. **g** Immunofluorescence showing the activation of M1 microglia (Red: Iba-1; Green: iNOS; Blue: DIPA; bar: 100 μm). **h** Iba-1and iNOS expression levels. **i** Western blot analysis of Nuc-Nrf2 in hippocampus. **j** Western blot analysis of Cyto-Nrf2 in hippocampus **k** Western blot analysis of HO-1 in hippocampus. Data are expressed as *mean ± SEM*, *n* = 6 /group

Previous studies have suggested that α7nAChR activator exerts its neuroprotective effect through activation of Nrf2/HO-1 signaling [22, 23]. To further confirm the effect of α7nAChR-mediated Nrf2/HO-1 pathway in sepsis, the potential anti-inflammatory mechanism of remimazolam was investigated via MLA and ML385 treatment. MLA or ML385 increased TNF-α and IL-6 expression levels in serum and hippocampus (Fig. 5a–d). Furthermore, MLA or ML385 decreased SOD content in hippocampus but increased MDA content (Fig. 5e, f). MLA or ML385 activated M1 microglia in CA1, DG, and cortex regions of the hippocampus while increasing the expression of Iba-1 and iNOS (Fig. 5g, h). Meanwhile, MLA or ML385 upregulated Nuc-Nrf2, while downregulating Cyto-Nrf2 and HO-1 (Fig. 5i–k).

### α7nAChR-mediated Nrf2/HO-1 Signaling Activation Exerts the Protective Effects on Cognitive Function

**Fig. 6:**
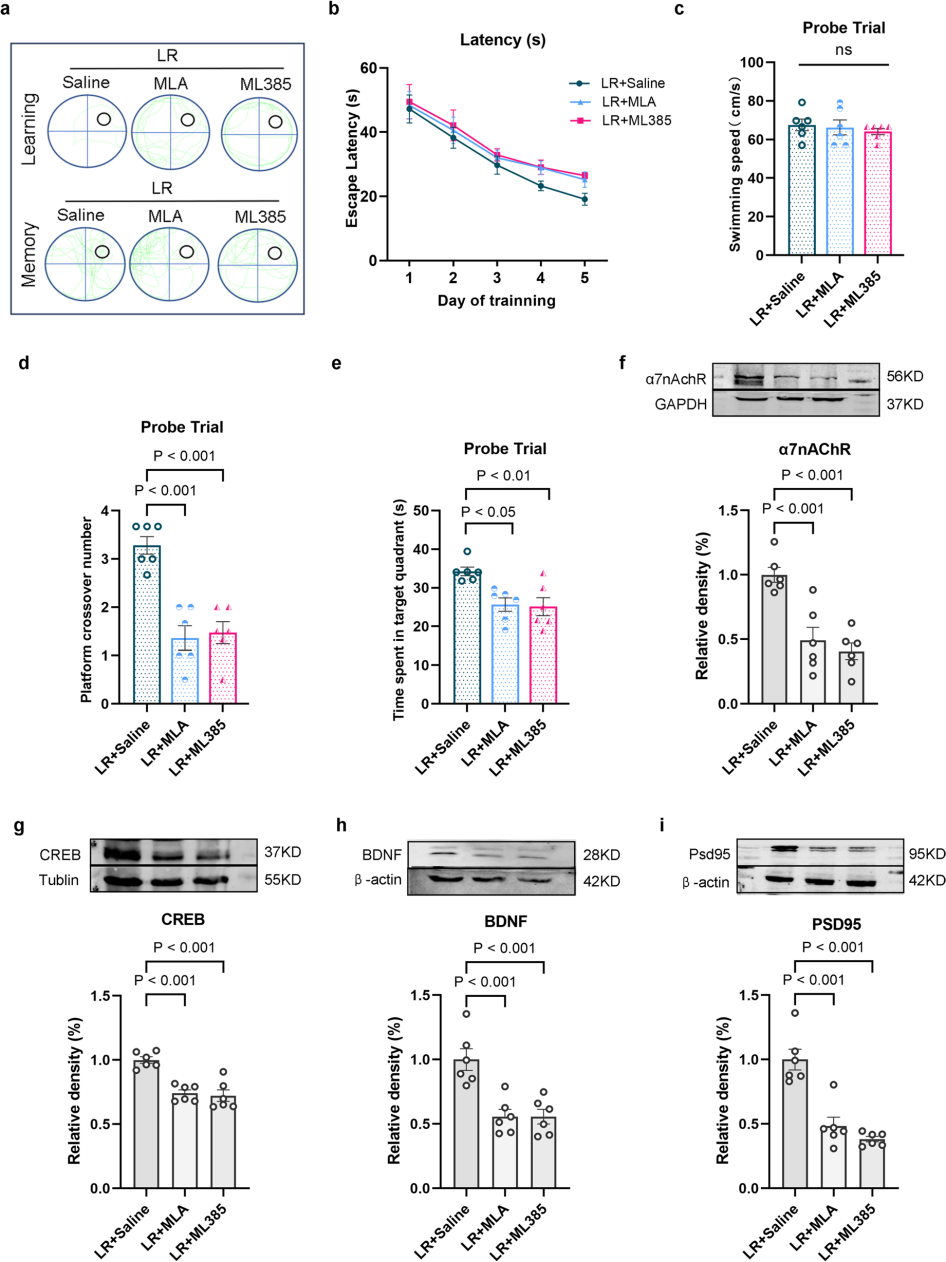
α7nAChR-mediated Nrf2/HO-1 signaling activation exerts the protective effects on cognitive function **a** The swim tracks of the rat during Morris water maze. **b** Escape latency to the platform during spatial working memory testing on days 1, 2, 3, 4 and 5. **c** Swimming speed during probe testing. **d** Platform-site crossings during probe testing. **e** The time traveled in the target quadrant during probe testing. **f** Western blot analysis of α7nAChR in hippocampus. **g** Western blot analysis of CREB in hippocampus. **h** Western blot analysis of BDNF in hippocampus. **i** Western blot analysis of PSD95 in hippocampus. Data are expressed as *mean ± SEM*, *n* = 6/group

Studies have shown that inflammatory response is closely associated with cognitive deficits [24]. The potential mechanism of remimazolam on cognitive was assessed via MLA and ML385 treatment, as shown by the learning and memory swimming traces during the Morris water maze (Fig. 6a). Escape latency increased during the positioning navigation trial over five consecutive days (Fig. 6b). However, swimming speed was not significantly different during the final (probe) trial (Fig. 6c), while the number of platform crossings and time spent in the target quadrant decreased (Fig. 6d, e). In addition, western blot analysis showed that α7nAchR and cognitive-related protein expression (CREB, BDNF, and PSD95) were downregulated (Fig. 6f–i). These findings indicate that remimazolam alleviates cognitive deficits via α7nAChR-mediated Nrf2/HO-1 signaling by attenuating neuroinflammatory response.

### The Roles of Subdiaphragmatic Vagus Nerve Integrity in the Anti-inflammatory Effects of Remimazolam

**Fig. 7:**
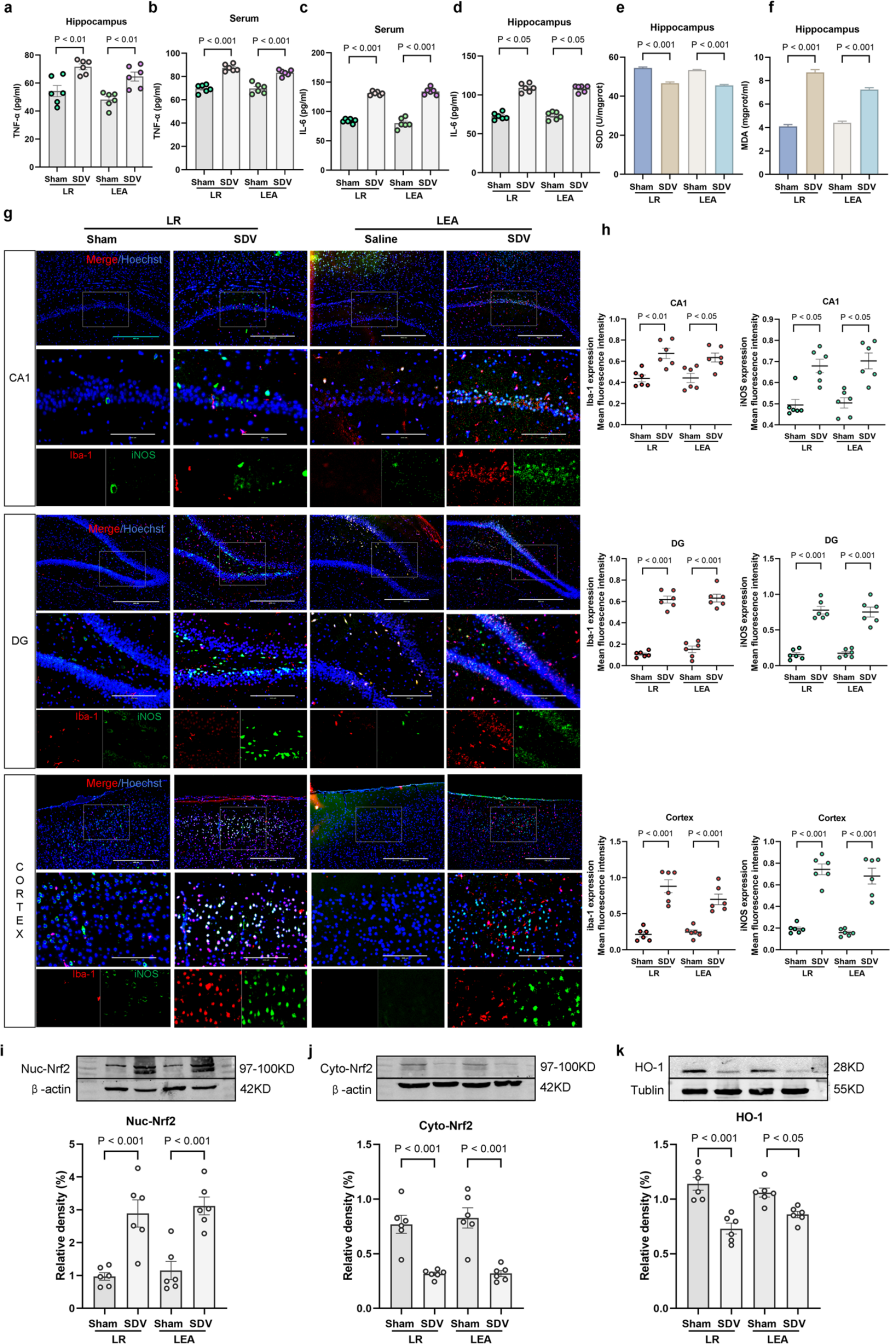
The roles of subdiaphragmatic vagus nerve integrity in the anti-inflammatory effects of remimazolam. **a** TNF-α expression in hippocampus. **b** Serum TNF-α levels **c** IL-6 expression in hippocampus. **d** Serum IL-6 levels. **e** SOD activity in hippocampus. **f** MDA content in hippocampus. **g** Immunofluorescence showing the activation of M1 microglia (Red: Iba-1; Green: iNOS; Blue: DIPA; bar: 100 μm). **h** Iba-1 and iNOS expression levels. **i** Western blot analysis of Nuc-Nrf2 in hippocampus. **j** Western blot analysis of Cyto-Nrf2 in hippocampus **k** Western blot analysis of HO-1 in hippocampus. Data are expressed as mean ± SEM, n = 6/group.

EA or remimazolam treatment after SDV operation up-regulated serum and hippocampal TNF-α and IL-6 levels (Fig. 7a–d), decreased SOD content, and increased MDA content (Fig. 7e, f). Furthermore, SDV operation enhanced M1 microglial activation in hippocampus, and up-regulated Iba-1 and iNOS (Fig. 7g, h). In addition, up-regulated Nuc-Nrf2 (Fig. 7i) and downregulated Cyto-Nrf2 (Fig. 7j) and HO-1 in hippocampus (Fig. 7k). These results suggest that SDV can abolish the anti-inflammatory protection effects of remimazolam. Remimazolam suppresses peripheral and central inflammatory responses through subdiaphragmatic vagus.

### The Role of Subdiaphragmatic Vagus Nerve Integrity in Remimazolam Rescue of LPS-Induced Cognitive Dysfunction

**Fig. 8:**
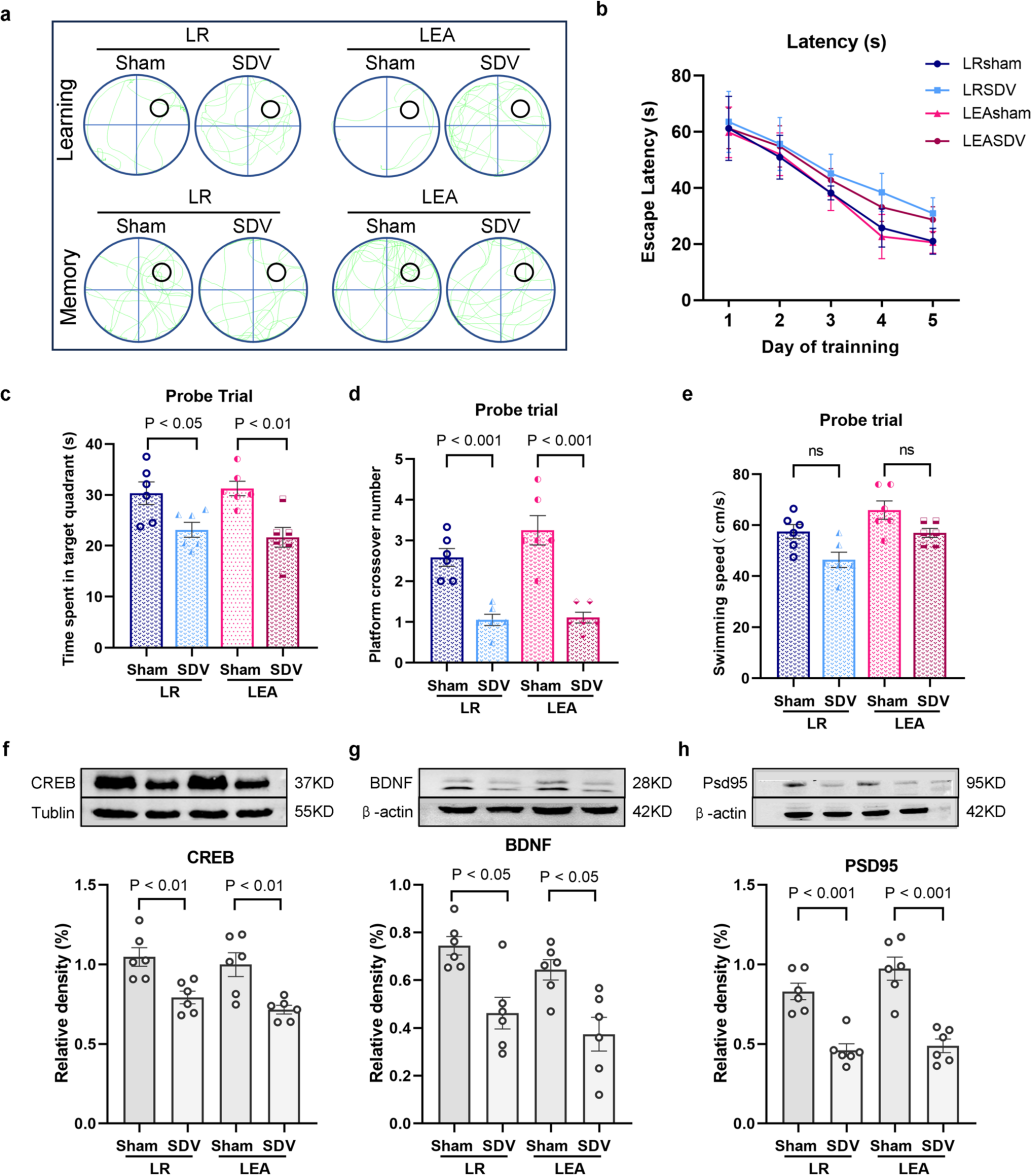
The role of subdiaphragmatic vagus nerve integrity in remimazolam rescue of LPS-induced cognitive dysfunction. **a** The swim tracks of the rat during Morris water maze. **b** Escape latency to the platform during spatial working memory testing on days 1, 2, 3, 4 and 5. **c** Swimming speed during probe testing. **d** Platform-site crossings during probe testing. **e** The time traveled in the target quadrant during probe testing. **f** Western blot analysis of CREB in hippocampus. **g** Western blot analysis of BDNF in hippocampus. **h** Western blot analysis of PSD95 in hippocampus. Data are expressed as *mean ± SEM*, *n* = 6/group

EA or remimazolam treatment after SDV operation, as shown by learning and memory swim traces in the Morris water maze (Fig. 6a), increased the escape latency during the positioning navigation trial for five consecutive days (Fig. 6b). However, swimming speed was not significantly different during the trial (Fig. 6c). Nonetheless, SDV surgery decreased the number of platform crossisng (Fig. 6d) and the time spent in target quadrant (Fig. 6e). Meanwhile, western blot analysis showed that SDV surgery down-regulated cognitive-related protein (CREB, BDNF, PSD95) expression levels (Fig. 6f–h), indicating that SDV surgery significantly weaken the therapeutic effect of remimazolam in LPS-challenged rats surgery (Fig. 8).

## Discussion

In the present study, LPS-induced systemic inflammation rat model was used to investigate the therapeutic effect of remimazolam in sepsis-induced cognitive dysfunction. Remimazolam ameliorated systemic inflammation and neuroinflammation(via vagus nerve target α7nAChR, activated Nrf2/HO-1 signaling pathway), decreased M1 microglial activation and oxidative stress in hippocampus, and reversed the decrease in dendritic branches and spine density, thus alleviating sepsis-induced cognitive dysfunction.

Systemic inflammation and chronic inflammatory state significantly promote cognitive decline and cognitive dysfunction in the brain [25]. Herein, immunostaining of a marker for microglia was higher in brain tissues of patients who died of sepsis, supporting that neuroinflammation occurs after sepsis onset. Sustained microglia activation enhances the production of inflammatory cytokines and reactive oxygen species (ROS), thus increasing neuronal apoptosis [26]. Synaptic dysfunctions and cognitive impairments, such as memory loss, are induced by ROS-mediated pathway [27]. Our previous study have shown that remimazolam has protective effects for cerebral I/R injury in rats by reducing neuronal injury and cerebral infarct and improving neurological function [8]. In this study, remimazolam suppressed neuroinflammation, oxidative stress, and microglia activation, thus alleviating LPS-induced cognitive dysfunction.

Souza showed that LPS can downregulate α7nAChR and exacerbate neuroinflammation, leading to cognitive dysfunction. However, activation of α7nAChR inhibits LPS-induced TNF-α release from neuroglia [21]. Furthermore, AChE inhibitors can improve the learning and memory behavior [28, 29]. Herein, remimazolam, EA, or PNU282987 reversed LPS-induced downregulation of α7nAChR and alleviated sepsis-induced cognitive dysfunction. Studies have shown that α7nAChR agonist, PNU282987, can enhance neuronal ROS generation and apoptosis after oxygen-glucose deprivation (OGD) treatment in hippocampal slices via activation of Nrf2/HO-1. However, microglia depletion can decrease the above neuroprotective effect [30]. Cao also indicated that activation of Nrf2/HO-1 pathway can attenuate cognitive dysfunction [31]. Nrf2 knockout can inhibit anti-inflammatory effects and downregulate α7nAChR. Furthermore, HO-1 knockout inhibits neuroprotective and antioxidant effects in mice [22]. Therefore, the α7nAChR-mediated Nrf2/HO-1 pathway significantly promotes the neuroprotective effects. In this study, remimazolam treatment up-regulated α7nAChR and decreased inflammatory cytokine, indicating that remimazolam can activate α7nAChR/Nrf2/HO-1 signaling as a protective measure. These results are consistent with the morphological and functional changes in rat neurons, suggesting that activation of α7nAChR/Nrf2/HO-1 by remimazolam inhibits microglia activation, attenuates systemic inflammation and neuroinflammatory stress, thus alleviating cognitive dysfunction and improving synaptic function.

α7nAChRs can regulate inflammation via a cholinergic anti-inflammatory pathway in microglia, thus attenuating neuronal loss and improving LPS-induced cognitive dysfunction [32, 33]. Vagal stimulation attenuates sepsis-induced peripheral inflammation and neuronal inflammation by activating afferent nerve fibers [34]. Therefore, vagal stimulation, as a surrogate marker or regulator of the neuroimmune axis, can be used for the treatment of sepsis-induced brain dysfunction. Similarly, our results have shown that vagal nerve transection inhibits the protective effect of remimazolam. Therefore, α7 nAChR regulation can be used for the treatment of CNS disorders, and one of the overlapping targets may be α7nAChR, which are cholinergic anti-inflammatory and pro-survival in intracellular pathways.

Organ preservation during perioperative or ICU sedation is crucial during anesthesia and critical care. Remimazolam has promising clinical potential and specific advantages over GABA receptor agonists [8]. Therefore, the sepsis-protective effect of remimazolam should be assessed to guide its use in perioperative or ICU sedation. Kinde [35] showed that the anesthetic action in the central nervous system may be due to the anesthetic binding to ligand-gated ion channels (LGICs). LGICs superfamily has many members, including γ-aminobutyric acid (GABA), glycine, 5-hydroxytryptamine 3 (5-HT3), and nicotinic acetylcholine receptors (nAChR). Down-regulated of α7nAChR impairs learning or concentration [36]. Propofol (100 µM), gamma-aminobutyric acid (GABA) receptor agonist, can positively regulate α7nAChR, otherwise it turns into an inhibitor at 300 µM or higher concentrations [37]. In this study, molecular docking experiments showed that the binding between Remimazolam and α7nAChR was relatively stable. However, α7nAChR inhibitors inhibited the protective effect of remrazolam.

In the present study, remimazolam ameliorated systemic inflammation and neuroinflammation (via vagus nerve target α7nAChR), activated Nrf2/HO-1 signaling pathway, inhibited M1 microglial activation, and reversed sepsis-induced cognitive dysfunction. Although cholinergic anti-inflammatory pathway plays an important role in the treatment of sepsis-induced cognitive dysfunction. However, this study has some limitations. First, only a single dose-remimazolam was introduced in the experiment. Besides, microglial type conversion and microglial energy metabolism mechanisms were not explored. Therefore, further research should assess the appropriate remimazolam doses and microglial-type conversion molecular mechanisms.

## Conclusions

Remimazolam induces a protective effect on sepsis through the cholinergic anti-inflammatory pathway of α7nAChR-mediated Nrf2/HO-1 signaling pathway. Furthermore, inhibitors and EA or SDV treatment improved the scientific structure and credibility of this study. However, further studies should assess the microglial-type conversion mechanisms in sepsis.

## Data Availability

The original data and materials in the study are included in this article. For further consultation, please contact the corresponding author.
